# Gene expression profiles in dental follicles from patients with impacted canines

**DOI:** 10.1007/s10266-018-0342-9

**Published:** 2018-02-12

**Authors:** Pamela Uribe, Lena Larsson, Anna Westerlund, Maria Ransjö

**Affiliations:** 1 Department of Orthodontics, Institute of Odontology, Sahlgrenska Academy, University of Gothenburg, PO Box 450, 405 30 Gothenburg, Sweden; 2 Department of Periodontology, Institute of Odontology, Sahlgrenska Academy, University of Gothenburg, Gothenburg, Sweden

**Keywords:** Connexin 43, Dental follicle, Gene expression, Impacted canines, Root resorption

## Abstract

Animal studies suggest that the dental follicle (DF) plays a major role in tooth eruption. However, the role of the DF during tooth impaction and related root resorptions in adjacent teeth is not clear. The hypothesis for the present study is that expression of regulatory factors involved in the bone remodelling process necessary for tooth eruption may differ between dental follicles from teeth with different clinical situations. We have analysed the gene expression profiles in the DF obtained from impacted canines, with (*N* = 3) or without (*N* = 5) signs of root resorption, and from control teeth (normal erupting teeth, mesiodens) (*N* = 3). DF from 11 patients (mean age: 13 years) obtains at the time of surgical exposure of the tooth. Due to the surgical time point, all teeth were in a late developmental stage. Gene expression related to osteoblast activation/bone formation, osteoclast recruitment and activation was analysed by RTqPCR. Genes related to bone formation (*RUNX2, OSX, ALP, OCN, CX43*) were highly expressed in all the samples, but osteoclast recruitment/activation markers (*OPG, RANKL, MCP*-*1, CSF*-*1*) were negligible. No apparent patterns or significant differences in gene expression were found between impacted canines, with or without signs of root resorption, or when compared to control teeth. Our results suggest the DF regulation of osteoclastic activity is limited in the late pre-emergent stage of tooth development, irrespective if the tooth is normally erupting or impacted. We suggest that the follicle may have an important regulatory function for alveolar bone formation in the final eruption process and CX43-gap junction communication could be an important signalling pathway.

## Introduction

Teeth are considered to be impacted when tooth eruption is delayed significantly and there is no clinical or radiographic evidence that further eruption will take place [1]. The maxillary permanent canine is impacted in 1–3% of most populations [1–3]. The significance of various clinical variables has been discussed in relation to the occurrence of impaction. However, using a comprehensive multivariate data analysis, we could not demonstrate any predictive correlation between impaction of the maxillary canines and any of the clinical variables examined [4]. This strongly suggests that other factors and altered regulatory mechanisms contribute to impaction of the maxillary canines.

Pioneering studies in animals conducted by Cahill and Marks [5, 6] and Wise [7] have demonstrated that the dental follicle (DF), i.e., the connective tissue sac surrounding an unerupted tooth, is essential for the intra-osseous phase of tooth eruption. Studies of the tooth eruption process in dogs have shown that it is accomplished through time-dependent resorption and formation of alveolar bone at specific sites around the erupting tooth [8]. This localised bone re-modelling seems to be regulated by the DF through a chronological and spatial expression of genes that are critical for osteoclast and osteoblast formation [9]. The eruption pathway comprises the resorption of alveolar bone (and the roots of the deciduous teeth), followed by bone formation in the base of the crypt [10]. The responsible cells, osteoclasts and osteoblasts, respectively, are activated by gene-regulated signals that are expressed in the DF.

The processes of recruitment and activation of osteoclasts are crucially dependent on receptor activator of nuclear factor kappa-B ligand (RANKL), produced by osteoblasts in response to various factors, e.g., hormones, prostaglandins, and pro-inflammatory cytokines [11]. Osteoprotegerin **(**OPG), which is produced by several cell types including osteoblasts and bone marrow stromal cells, is a decoy receptor that binds to the RANK receptor on osteoclast precursors and mature osteoclasts. The RANKL/OPG ratio determines osteoclast recruitment and bone resorption (for details on osteoclasts and their regulation and functions, see Kular et al. 2012, Martin and Sims 2015) [12, 13]. The differentiation of osteoblasts is regulated by several factors, e.g., bone morphogenetic proteins (BMPs), which bind to specific receptors and activate downstream specific transcription factors, such as RUNX2 (alias cbfa1) and osterix (OSX). The activities of alkaline phosphatase (ALP) and the matrix proteins produced by osteoblasts, such as collagen type I and osteocalcin (OCN), are important for the bone formation process [14].

Earlier studies on tooth eruption in dogs and rats revealed the expression of colony-stimulating factor 1 (CSF-1) [15], monocyte chemoattractant protein-1 (MCP-1) [16], RANKL [17], OPG [18], and BMP2 [9] in the DF. Since the genes required for tooth eruption are expressed in the DF, tooth impaction may be related to the signals that are regulated by the follicle [5]. The concept of a genetic molecular cause for tooth eruption failures is not new [19]. The eruption failure observed in cases of cleidocranial dysplasia has been related to the altered expression of RA*NKL/OPG* in the DF [20]. Furthermore, familial cases of primary failure of eruption (PFE) have been linked to a defect in the gene for parathyroid hormone receptor 1 (*PTH1R*) [19, 21]. Since tooth eruption depends on the DF, studies on the gene expression of impacted teeth may shed some light on the mechanism behind aberrant eruption.

Tooth eruption in humans is difficult to study, primarily because it occurs so slowly and the teeth are so inaccessible until they emerge into the mouth. As a result, neither the eruption mechanism nor the controlling factors in human tooth eruption are completely understood [22]. A clinical complication that is commonly seen with impacted maxillary canines is the occurrence of root resorptions in the adjacent teeth. Using cone beam computed tomography, the root resorption was estimated to occur in 23–50% of the incisors adjacent to the impacted canines [23, 24]. Close proximity of the impacted tooth and adjacent root increases the risk of root resorptions [25]. This suggests that root resorption is caused by osteoclast-regulating factors that are expressed by the DF. The gene expression patterns of the DF from impacted canines have not been analysed previously in relation to the clinical signs of root resorption.

This study is based on the hypothesis that the expression of regulatory factors involved in the bone remodeling process necessary for tooth eruption differs between dental follicles from control teeth and impacted teeth from different patients and with contrasting clinical situations. The aim was, therefore, to analyse the gene expression profiles of the DF from impacted canines, with or without signs of root resorption, and from controls (normal erupting teeth and mesiodens), to investigate the eruption mechanism and potential differential expression of regulatory factors.

## Materials and methods

### Ethics

This study was approved by the Regional Ethics Board at the University of Gothenburg (Dnr. 898-13) and by the National Data Inspection Board. Informed consent was obtained from all individual participants included in the study.

### Sampling

**Fig. 1:**
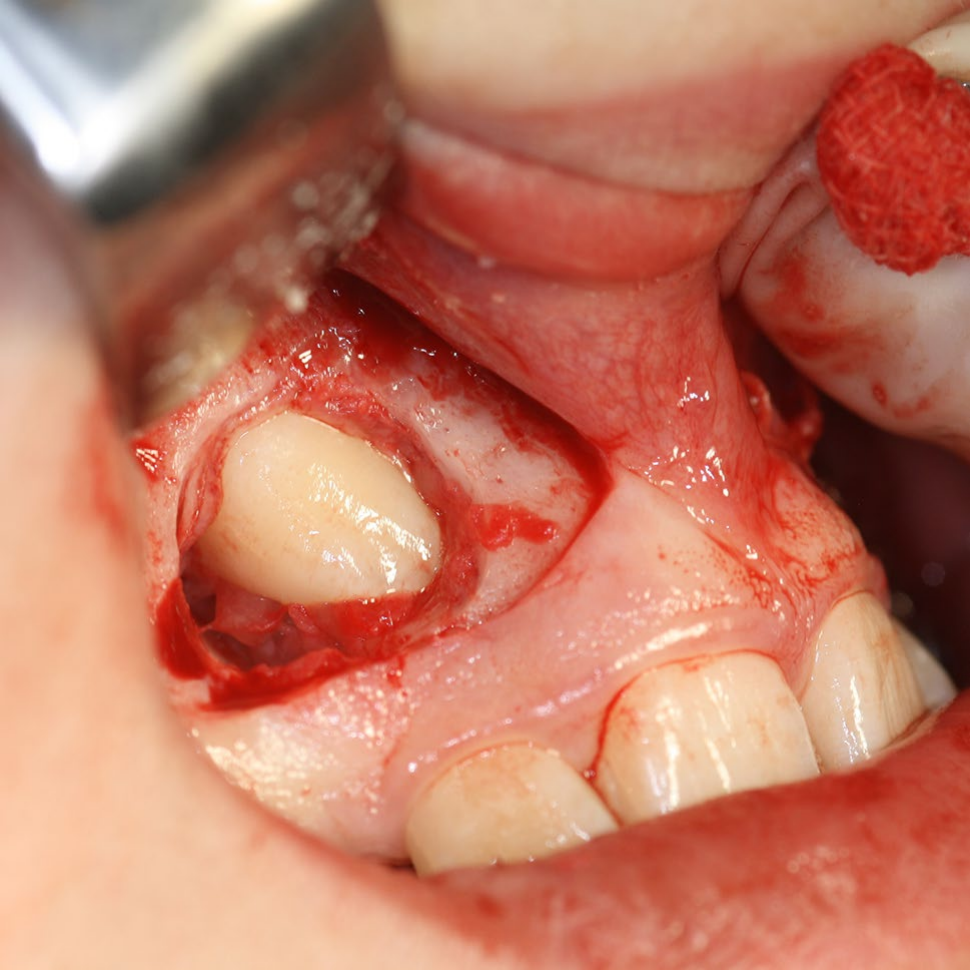
Illustration of the sampling process. Dental follicles were isolated during surgical exposure of impacted teeth

**Fig. 2:**
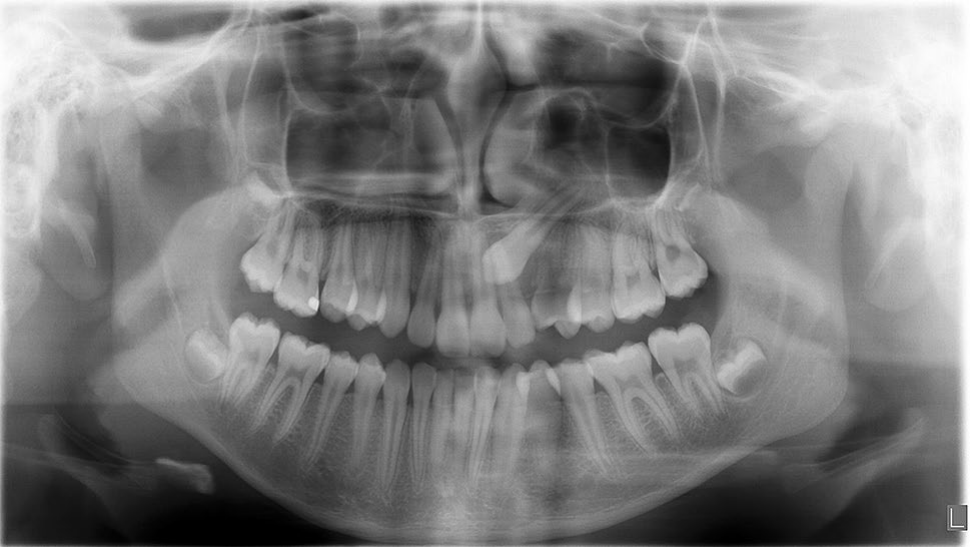
Radiograph from a patient with unilateral maxillary canine impaction. Panoramic radiograph showing root length and degree of development of an impacted maxillary canine

Samples of the DF were collected during surgical exposure of impacted teeth (Fig. 1). All operations were performed at the Clinic of Oral and Maxillofacial Surgery and the Clinic of Pedodontics at Mölndal Hospital, Mölndal, Sweden. Six females and five males (mean age 13 years; age range 10–16 years) were enrolled. In total, 11 DF from permanent teeth were collected: 8 from permanent maxillary impacted canines, 2 from mesiodens; and 1 from a normally erupting tooth (Table 1). All teeth were intra-osseous and entirely covered by bone. All the teeth had considerable root length and were recorded in the ending developmental stages (G-H) according to Demirjian’s dental age assessment [26]. No gingival tissue was attached to the collected specimens. Patients with craniofacial syndromes, systemic metabolic diagnosis or any bone disease were excluded from the study. Impaction was decided when eruption is considerable delayed, and for which there is clinical and radiographic evidence that further eruption may not occur within the normal period of growth (Fig. 2). For unilateral impaction, the eruption of the contra-lateral tooth was always used as a control. In case where bilateral impaction occurred, developmental stages of the bite were always taken into account. The rationale for comparing the levels of regulatory factors with mesiodens is that these excess teeth rarely cause root resorptions, even though they are often located in close proximity to the roots of the adjacent incisors.

**Table 1:** Description and distribution of the patients’ samples

Sample	Tooth	Gender	Age	Root resorption in adjacent tooth	Position
1	13	Male	14	No	Palatal
2	Mesiodens	Male	14	No	Palatal
3	13	Female	12	No	Buccal
4	13	Female	11	Yes	Buccal
5	23	Female	11	Yes	Buccal
6	13	Female	15	No	Palatal
7	13	Female	13	Yes	Buccal
8	23	Male	15	No	Palatal
9	45	Male	16	No	Lingual
10	Mesiodens	Male	10	No	Palatal
11	23	Female	13	No	Palatal

In total, 11 samples were collected from 9 patients. Samples highlighted in grey are from the same patient. Root resorption of the adjacent lateral incisor was determined by clinical and radiographic examinations

Immediately after their removal, the DF was placed in PAXgene^®^ tissue containers (PreAnalityx, Hombrechtikon, Switzerland). PAXgene^®^ is a formalin-free system that is designed to improve the quality of molecular analysis without diminishing the quality of downstream and histopathological analyses. They are dual-cavity containers prefilled with fixation and stabiliser reagents. The specimens were fixed in PAXgene Tissue FIX Solution for 2–4 h, and then transferred to the PAXgene^®^ tissue STABILIZER solution in the same container. Specimens were stored at − 80 °C until used.

### Histology

Dental follicle specimens were fixed in PAXgene^®^ tissue container solutions (PreAnalityx). One half of the specimens was sent for standard embedding and sectioning to Biobanken Norr (Umea, Sweden). Fixed tissues were placed in embedding cassettes, dehydrated, and embedded in paraffin. Serial sections of 4-µm thickness were cut at three different levels, and split onto three separate slides. Haematoxylin and Eosin staining was used to visualise the morphology. Briefly, sections were placed at 60 °C for 1 h until the paraffin was melted. After rehydration, the sections were submerged in Mayer’s haematoxylin for 30 min and rinsed under tap water for 30 min. Eosin was added for 30 s, and the sections were then washed with dH_2_O. Microphotographs were taken using an Olympus BX43 microscope and an Olympus UC30 camera under 10×magnification. For imaging processing, the CellSense software (Olympus Corp., Tokyo, Japan) was used.

### Real-time quantitative polymerase chain reaction (RT QPCR)

The Minimum Information for Publication of Quantitative Real-Time PCR Experiments (MIQE) guidelines was followed to ensure the relevant experimental conditions and assay characteristics [27]. The remainder of the specimens was used for downstream analyses. RNA extraction was carried out using the commercially available PAXgene Tissue RNA Kit (Qiagen, Hilden, Germany) according to the manufacturer’s instructions. Dental follicles were homogenised in the lysis buffer using a gentle MACS dissociator (Miltenyi Biotech, Bergish Gladbach, Germany). DNase I treatment was performed to remove any contaminating DNA. The RNA concentrations were quantified with the Qubit™ Fluorometer (Invitrogen, Burlington, Ontario, Canada) and stored at − 20 °C.

All reverse transcription steps were performed using the iScript™ cDNA Synthesis Kit (Bio-Rad Laboratories, Hercules, California, USA) with 1 μg of total RNA. Two microliters of Universal RNA Spike (TATAA Biocenter, Gothenburg, Sweden) were added to each sample to allow quality control throughout the entire RT qPCR experimental workflow.

To select the most stable reference genes for normalisation, a panel of twelve reference genes was screened in five samples from the retrieved tissues. The expression profiles of the screened reference genes were evaluated using the geNorm [28] and Normfinder [29] programmes.

The primers used in the RT-qPCR were purchased from Bio-rad Laboratories and are listed in Table 2. The analysis of the target genes and the best two selected reference genes was performed in a 10-μl reaction volume (10 ng of cDNA per reaction) in duplicate on a CFX 96 Real-Time System (Bio-Rad Laboratories) using the SsoAdvanced™ Universal SYBR^®^ Green Supermix (Bio-Rad Laboratories). An inter-plate calibrator (TATAA Biocenter) was added to each plate to compensate for the variation between runs. The quantities of the target genes were normalised using the geometric mean of the Cq values of the selected reference genes. The normalised relative quantities were calculated using the delta–delta Cq method and 90% PCR efficiency [30].

**Table 2:** Biorad SYBR^®^ green primers used for the RT-qPCR

Gene identification	Abbreviation	Unique Assay ID
Target gene encoding:		
Runt-related transcription factor 2	RUNX2	qHsaCED0044067
Sp7 transcription factor	OSX	qHsaCED0003759
Bone morphogenetic protein 2	BMP2	qHsaCID0015400
Alkaline phosphatase	ALP	qHsaCID0010031
Bone gamma-carboxyglutamate protein (BGLAP)	OCN	qHsaCED0038437
Gap junction protein, alpha 1, 43 kDa	CX43	qHsaCID0012977
Tumour necrosis factor receptor superfamily, memb.11b	OPG	qHsaCED0046251
Tumour necrosis factor (ligand) superfamily, memb. 11	RANKL	qHsaCID0015585
Chemokine (C–C motif) ligand 2 (CCL2)	MCP-1	qHsaCID0011608
Colony-stimulating factor 1 (macrophage)	CSF-1	qHsaCID0016847
Reference gene encoding		
Glucuronidase, beta	GUSB	qHsaCID0011706
Hypoxanthine phosphoribosyltransferase 1	HPRT1	qHsaCID0016375

### Statistical analysis

The gene expression data were analysed using the PRISM 7 software package (GraphPad Software Inc., San Diego, California, USA). Analysis of variance (ANOVA) was performed to test for significant differences in gene expression between follicles obtained from patients with different clinical situations, with statistical significance adjudged for *p*-values < 0.05.

## Results

### Histology

**Fig. 3:**
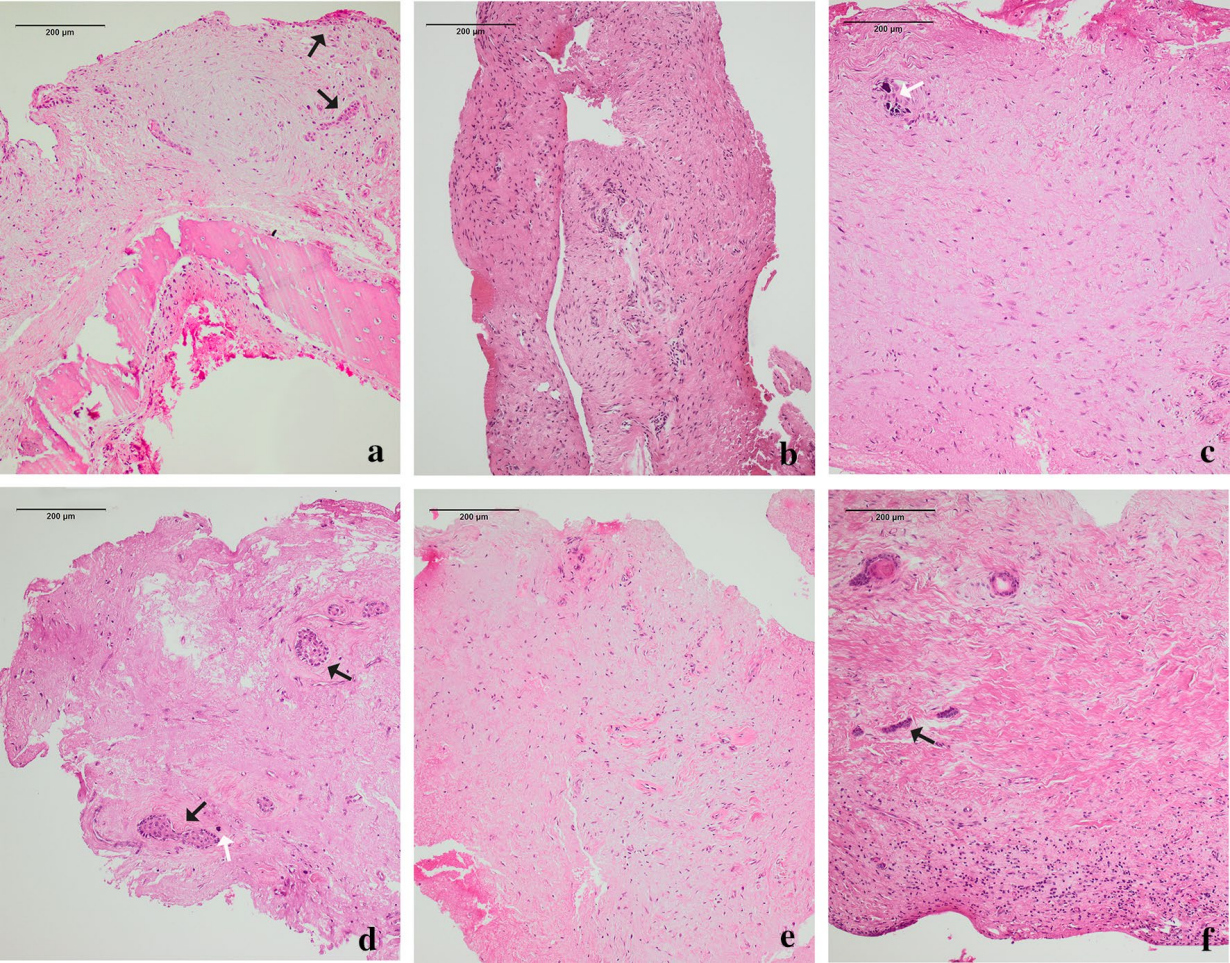
Micrographs of histological sections of dental follicles stained with Haematoxylin and Eosin. **a** Patient 1, tooth 13, without resorption activity in the neighbouring teeth; **b** Patient 2, mesiodens; **c**–**d** Patient 3, tooth 13, no resorption activity in the neighbouring teeth, showing different areas from the same specimen; **e** Patient 5, tooth 23, *with* signs of resorption in the root of the lateral incisor; **d** Patient 9, tooth 45, without resorption activity in the neighbouring teeth

Microscopic examination of histological sections produced from the DF of the impacted canines and control teeth revealed a relatively heterogeneous morphology, both within the samples and between samples (representative photomicrographs shown in Fig. 3 a–f). The major part of the tissue sections consisted of fibrous connective tissue with sparsely scattered fibroblast-like cells, as well are areas that contained more cells. Islands and strands of odontogenic epithelium were observed in most of the sections (black arrows). Small pieces of bone tissue and more diffuse calcifications were also evident in some specimens (white arrows). No consistent pattern was detected for the appearance of the tissue sections in relation to clinical signs of resorption or lack thereof in the roots of the neighbouring teeth.

### Gene expression analysis

To examine the possible differential regulatory capacity of the DF, gene expression analysis was employed and comparisons were made of the gene expression profiles of the different samples. The reference gene, *GUSB* and *HPRT1*, showed the most stable gene expression between the samples and was, therefore, selected as reference genes for the present analyses. The measured Cq value and the shape of the amplification curve reflected no inhibition in the presence of RNA spiking in the control assays.

To facilitate the interpretation of the RT-qPCR results within the different samples, we present the scaled gene expression ratios of the target genes to the reference gene *GUSB* (Table 3). Warm colours (red) indicate higher expression levels, and cold colours (blue) indicate low expression levels.

**Table 3:**
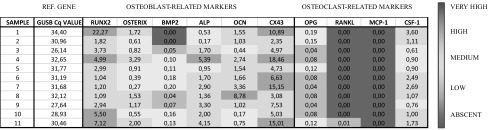
Scaled gene expression ratios of target genes related to reference gene

The gene expression levels are adjusted by standardisation based on the expression levels of *GUSB*. A very high expression level is adjudged as a > 5-fold increase; high is a 1–5-fold increase; medium is a 0.1–1.0-fold increase; and low is defined as a 0.0–0.1-fold fold increase in expression

**Fig. 4:**
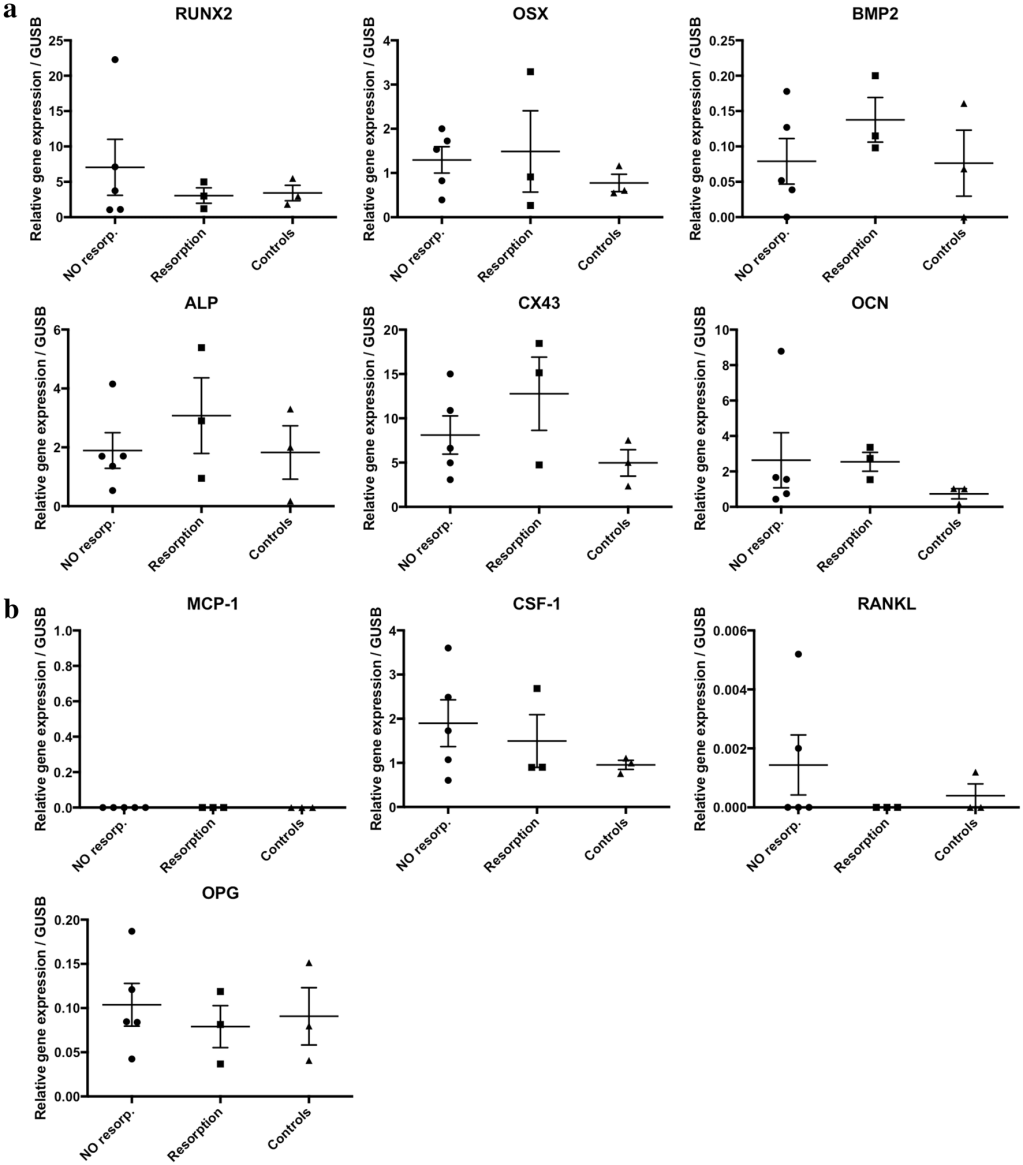
Comparison of gene expression profiles of dental follicles from impacted teeth with different clinical situations. Relative expression levels of the indicated target genes related to markers of bone formation (**a**) and bone resorption (**b**) in the DF from impacted teeth. The data are presented as delta–delta Cq values in relation to the relative gene expression level of the selected reference gene (*GUSB*). The horizontal lines indicate the mean values and the vertical lines represent the SEM. Symbols used: the circles represent the five specimens from impacted canines without any resorbing signs in the adjacent roots of the laterals; the squares represent the three samples from impacted canines with clinical and radiographic signs of root resorption in the roots of the adjacent laterals; and the triangles represent the control teeth comprising two mesiodens and a normally erupting tooth

As shown in Table 3 and Fig. 4, the highest levels of expression were for genes involved in bone formation, namely *RUNX2* and *CX43*. Relatively high gene expression levels were seen in all the samples for *OSX*, *ALP*, and *OCN* whereas the *BMP2* gene showed very low expression in all the samples. The expression levels of *CSF*-*1*, *RANKL* and *OPG*, which are markers related to osteoclast recruitment, were low in all the specimens. *MCP*-*1* expression was absent in every sample. There was a relatively large variation in the expression levels for all genes that were expressed, with the exception of the *CX43* gene, which was strongly expressed in all the evaluated specimens in relation to the expression level of the reference gene.

No significant difference or any clear pattern was observed when comparing the follicles and relating their gene expression levels to their different clinical situations (root resorptions, impaction, normal eruption).

## Discussion

In the present study, we have analysed the gene expression profiles of selected critical gene markers in human DF from impacted maxillary canines, mesiodens, and a normally erupting premolar, and investigated the correlation with root resorption of adjacent teeth.

Our results reveal that genes related to bone formation had high expression levels in all the samples, while the expression levels of markers for osteoclast recruitment and activation were negligible. No apparent patterns or significant differences in the mRNA expression of marker genes were identified between impacted canines, with or without clinical signs of root resorption, or compared to the follicles from mesiodens or the normally erupting premolar.

The advantage of using animal models when analysing the molecular regulation of cellular activities in the DF is the possibility to perform chronological studies with precise time-points and at specific locations. Obviously, it is not possible to obtain such time- and site-specific samples from patients. Thus, a limitation in the present study is that the clinical situation and the day of surgery dictated the time-point, as well as the sampling site in the follicle.

Our results, in relation to markers of bone formation in the human DF, are in agreement with previous animal experimental studies. The relatively high expression levels of *RUNX2*, *OSX*, *ALP*, and *OCN* in all our samples confirm the capacity for osteogenic regulation in human DF. The protein expression levels have been confirmed with immunofluorescence staining (data not shown). Furthermore, these results strongly suggest that the DF is actively regulating osteogenesis when teeth are in a late pre-eruptive stage.

The mechanism or motive force that drives the tooth through the resorbed alveolar bone has not been clearly defined, although many studies have shown that bone growth at the base of the tooth crypt is required for a tooth to erupt [31–33]. Our data suggest that the dental follicle is actively regulating osteogenesis when the teeth are in a late pre-eruptive stage. Previous studies have demonstrated that inhibition of members of the BMP superfamily can disrupt alveolar bone formation and, subsequently, tooth eruption, despite the fact that an eruption path is formed [33]. Therefore, it appears that *RUNX2, OSX,* and *BMP2* are key molecules in promoting osteogenesis during the eruption process. The strong expression of the gene for gap junction protein CX43 in all the samples is interesting, considering that many regulatory signals between cells in developmental processes are mediated by gap junctional transport of ions, small molecules, and second messengers. Gap junction channels between two docking cells are formed by transmembrane CX proteins, of which CX43 is the most common in bone cells [34]. CX43-gap junction communication is crucial for development of osteoblasts as well as osteogenesis and craniofacial bone development [35]. In addition, osteoclast precursors and mature osteoclasts are reported to express the CX43 protein and functional gap junction channels necessary for their development and bone-resorbing activities [36–38]. However, considering the low-level expression for osteoclast activity in the present samples, we conclude that the high expression of *CX43* in the dental follicle cells more likely reflects an osteoblast differentiation process. Thus, signalling pathways that involve gap junction communication may be involved in the DF regulation of local bone formation around the erupting tooth.

While bone-resorbing osteoclasts are required for tooth eruption, they are also involved in the root resorption process, which is often seen as a complication associated with impacted and displaced maxillary canines. The aetiology of root resorption is multi-factorial and possibly related to susceptible root areas. Nevertheless, since resorption is dependent upon local signals for the recruitment and activation of osteoclasts, it would be reasonable to assume that the DF may be involved in this. We have not analysed gene expression of osteoclast markers; we have only included the expression for factors regulating their recruitment and activation. The reason is the fact that recruited and activated osteoclasts are only found close to bone surfaces (or root), and thus not in follicle tissue itself.

In contrast to previous animal studies, all our samples showed low expression of genes related to regulation of osteoclast recruitment or activation (*CSF*-*1, MCP*-*1, RANKL* and *OPG*). The low levels of osteoclast-activating signals in the DF from impacted teeth and mesiodens might be attributed to their impaction condition. However, the expression of osteoclast-regulating factors was not higher in the DF from normally erupting teeth or impacted canines with clinical and radiographic signs of root resorption in adjacent teeth. The time factor could be crucial, since at the time of surgery the examined DF were from teeth that were in a late developmental stage, and probably with cessation of the intra-osseous eruption stage. Taking this together, our results strongly suggest that the DF does not regulate bone resorption in the final pre-emergent stages of eruption.

It could be argued that since the surgical procedure did not provide the complete follicles from the patients, there might be regional differences on the regulatory capacity of the DF, as indicated in the animal studies by Marks and Cahill [39]. We are currently investigating if there is a different regional expression of regulatory factors in human DF and, furthermore, if a differential capacity of stimulated expression of regulatory factors in DF cells may explain the differences in the contrasting clinical situations.

Only one previous study has analysed the expression of genes in the DF of human subjects related to the eruption process. They reported alterations in the expression of genes that encode regulators of osteoclastogenesis in subjects who were suffering from cleidocranial dysplasia, and had multiple unerupted supernumerary teeth [20]. While our results cannot prove the actual cause of impaction, they still provide more insight into the relatively unexplored field of tooth eruption in humans. Most of the earlier data on the molecular basis of tooth eruption were obtained from studies conducted with rodents and dogs. Extrapolation of these data to humans should be approached with caution given the known differences between the diverse species. Thus, the significance of the present work in relation to tooth eruption, and the disturbance of eruption that is inherent to impaction, pertains to the exploration of the gene expression profiles of human DF. Furthermore, the osteogenic capacity of the DF is intriguing and may be investigated further as a potential source for regenerative therapies.

## Conclusion

The role of the DF regulating osteoclastic activity is limited in the late pre-emergent stages of tooth eruption, irrespective if the tooth is normally erupting or impacted. The eruptive force in this final developmental stage is regulated by other tissues or molecular factors that warrant further investigation. However, the DF has an influential regulatory function for alveolar bone formation in late pre-emergent stage. CX43-gap junction communication in the DF may be an important signalling pathway in the final eruption process.
